# Supplementary figures and images for: Targeting enhanced digestibility: Prioritizing low pith lignification to complement low p-coumaric acid content as environmental stress intensity increases
Source: PLoS One. 2025 Dec 5;20(12):e0338077. doi: 10.1371/journal.pone.0338077 (PMC12680253; doi:10.1371/journal.pone.0338077)

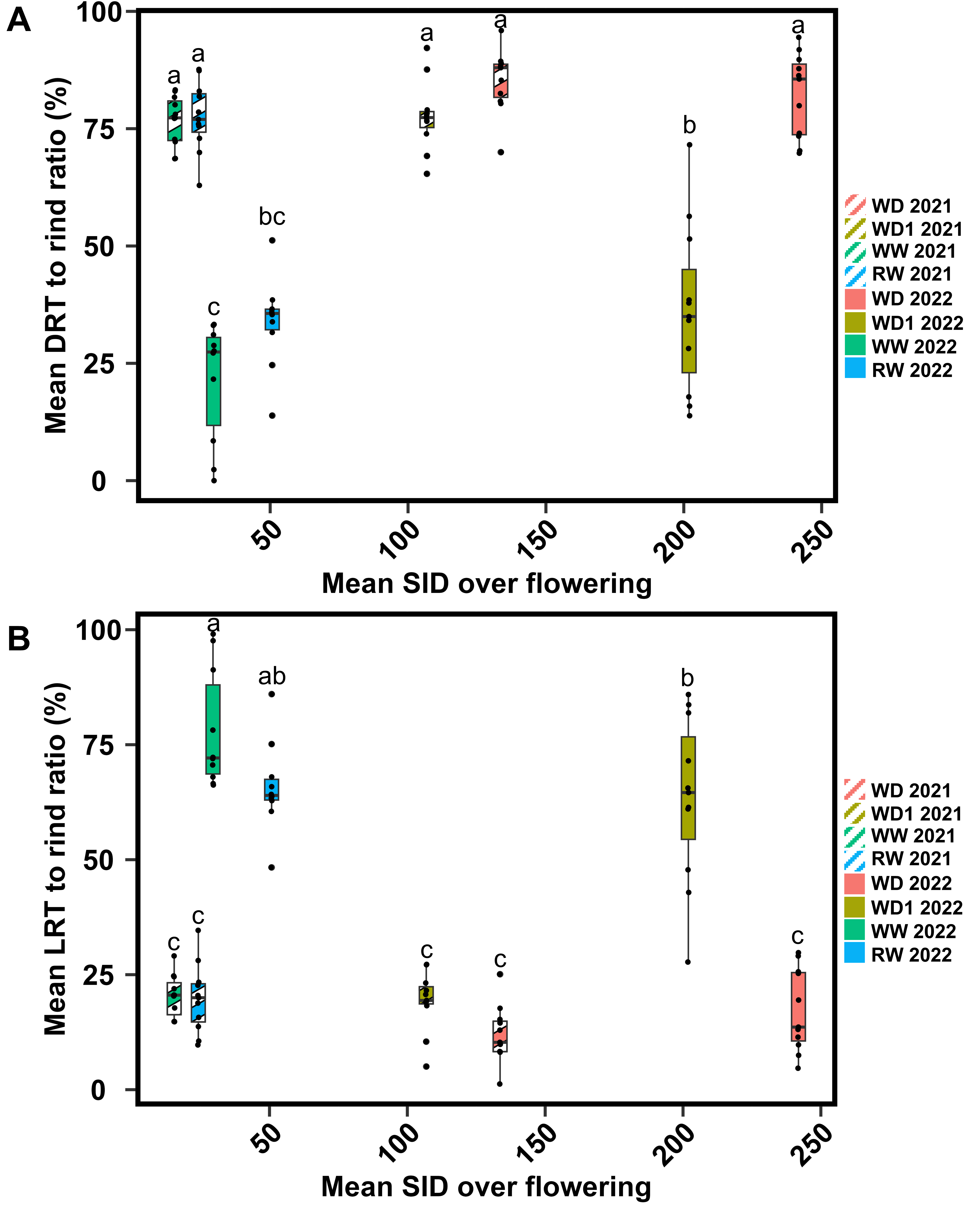

Supplement: S1 Fig — (A) DRT to rind and (B) LRT to rind ratios to the SID value measured across anthesis and silking (Pflo) per variety under the eight conditions. Letters on top of bars represent the Tukey class for the given condition (P < 0.05). (TIF) [file pone.0338077.s005.tif]
